# Independent predictive value of blood inflammatory composite markers in ovarian cancer: recent clinical evidence and perspective focusing on NLR and PLR

**DOI:** 10.1186/s13048-023-01116-2

**Published:** 2023-02-09

**Authors:** Chuan-long Zhang, Xiao-chen Jiang, Yi Li, Xue Pan, Meng-qi Gao, Yan Chen, Bo Pang

**Affiliations:** 1 Guang’anmen Hospital, China Academy of Chinese Medical Sciences, Beijing, 100053 China; 2 Wangjing Hospital, China Academy of Chinese Medical Sciences, Beijing, 100102 China; 3 International Medical Department of Guang’anmen Hospital, China Academy of Chinese Medical Sciences, Beijing, 100053 China

**Keywords:** Ovarian cancer, Composite inflammatory markers, Predictive value, Clinical evidence, NLR, PLR

## Abstract

Ovarian cancer (OC) is one of the deadliest malignant tumors affecting women worldwide. The predictive value of some blood inflammatory composite markers in OC has been extensively reported. They can be used for early detection and differential diagnosis of OC and can be used for predicting survival, treatment response, and recurrence in the affected patients. Here, we reviewed the predictive values of composite inflammatory markers based on complete blood count, namely neutrophil-to-lymphocyte ratio (NLR), platelet-to-lymphocyte ratio (PLR), monocyte-to-lymphocyte ratio, and systemic inflammation index and markers based on blood protein, namely C-reactive protein-to-albumin ratio and prognostic nutritional index in OC, with a focus on NLR and PLR. We referred to the clinical studies on these six markers, reviewed the patient population, and summarized the marker cut-off values, significance, and limitations of these studies. All these studies were retrospective and most of them were single-center clinical studies with small sample sizes. We found that the cut-off values of these markers have not been unified, and methods used to determine these values varied among studies. The predictive value of these markers on survival was mainly reflected in the postoperative patients of multiple subtypes of ovarian cancer including epithelial OC, high-grade serous ovarian carcinoma, and ovarian clear cell carcinoma. We focused on NLR and PLR and calculated their pooled hazard ratios. NLR and PLR were reliable in predicting overall and progression-free survivals in patients with OC. Therefore, it is necessary to adjust important confounding factors and conduct a long-term follow-up prospective cohort study to further clarify the cut-off values of NLR and PLR and their clinical applications.

**Supplementary Information:**

The online version contains supplementary material available at 10.1186/s13048-023-01116-2.

## Introduction

GLOBOCAN database (2020) included 313,959 new cases and 207,252 deaths for ovarian cancer (OC) [1]; these values were worse than those predicted in 2016 [2]. OC is the eighth most common cancer, accounting for 4.7% of all female cancer-related deaths and poses a serious threat to the lives and health of women worldwide [3]. OC is often regarded as a “silent killer” because of the lack of specific symptoms in the early stages and effective early diagnostic strategies [4]. Currently, surgery and platinum-based chemotherapy are the common treatment strategies for patients with OC [5, 6]. Although the initial remission rate of patients with OC is 60%–80%, 70% of patients with advanced OC relapse within 5 years of remission, and many acquire drug resistance [7, 8]. The five-year survival rate for patients with advanced ovarian cancer (stage III or IV) is < 30% whereas that for patients in early stages is 95% [9]. Survival prediction, early diagnosis, differential diagnosis, and treatment response prediction are required for patients with OC. Therefore, reliable markers are urgently required to guide the diagnosis and treatment options for patients and doctors.

The primary challenge is the diagnosis of OC, which includes differential diagnoses from other abdominal masses. Further, patients who receive chemotherapy may develop some adverse outcomes, such as drug resistance, recurrence, and death. Currently, we are unable to accurately predict the survival of patients with OC. Even before the occurrence of ovarian cancer, tumor cells induce the body to produce an environment suitable for tumor occurrence and development, including an inflammatory microenvironment. The British surgeon Stephen Paget first put forward the “seed-soil” theory, which laid the foundation for the concept of the tumor microenvironment. This is an extremely complex cellular network, in which a variety of inflammatory factors secreted by tumor and stromal cells (such as fibroblasts) construct an inflammatory microenvironment. This inflammatory microenvironment greatly affects the malignant characteristics of the tumors by regulating the biological processes involved in their development [10, 11]. This explains why even OC patients who are diagnosed and treated at early stages achieve poor outcomes. Therefore, evaluating inflammatory responses is crucial in the prognosis of OC. Uncontrollable inflammation plays an important role in inducing and promoting tumors, and the state of inflammation is reflected in the changes in blood inflammatory markers. Measurable parameters in the blood that reflect systemic inflammation include increased levels of leukocytes and their subtypes, elevated platelets, elevated C-reactive protein (CRP), and decreased albumin (ALB) [12, 13]. This evaluation approach is minimally invasive, low-cost, and easily available.

White blood cells are the largest group of inflammatory cells, and their several subtypes including neutrophils, lymphocytes, and monocytes are important inflammatory markers. Neutrophils can promote tumor progression by releasing tumor necrosis factors, interleukin-1 and interleukin-6 [14]. Lymphocytes are crucial in tumor-specific immune responses by inducing cytotoxic cell death and inhibiting the proliferation and migration of tumor cells [10]. Monocytes are involved in tumor occurrence, growth, migration, vascularization, invasion, and metastasis [15]. Therefore, white blood cells are considered the first traditional markers of inflammation. In recent years, the role of platelets as a marker of inflammation has been gradually recognized as they have distinct roles in inflammatory responses. Platelets can induce the epithelial–mesenchymal transformation of tumor cells in circulation and promote extravasation to the metastatic site [16]. Macrophages, neutrophils, and platelets present in the chronic inflammatory environment of tumors secrete cytokines, proteases, angiogenic factors, and chemokines [17]. Inflammation in cancer, caused by neutropenia, lymphopenia, monocytosis, and thrombocytosis, accelerates cancer progression by inducing angiogenesis, invasion, metastasis, and paraneoplastic phenomena [18–20]. In addition, some proteins in the blood, such as CRP and ALB, can reflect the inflammatory status of the body. These proteins have a certain correlation with the prognosis of OC [21]. However, a single blood parameter as an inflammatory marker does not reflect the state of inflammation; the values may represent secretion from tumor cells of an underlying infection. Therefore, it is complicated to evaluate the predictive values of such parameters in cancer. In addition, some currently used clinical indicators, such as the palliative prognostic index, play a good prognostic role and are easy to use without any invasive tests or the help of an experienced physician [22]. Yoshida et al. reported that cancer antigen 125 (CA125) is a better parameter than other complete blood count parameters for the prediction of ovarian tumors before surgery [23]. Therefore, the predictive value of inflammatory cells such as neutrophils and inflammatory proteins such as CRP is controversial in OC, and their use in clinical practice is not preferred.

Compared with single blood parameters as the inflammatory markers, the predictive value of blood inflammatory composite markers, such as neutrophil-to-lymphocyte ratio (NLR), platelet-to-lymphocyte ratio (PLR), monocyte-to-lymphocyte ratio (MLR), systemic inflammation index (SII), C-reactive protein albumin ratio (CAR), and prognostic nutritional index (PNI), is better because of their sensitivity and stability. They have been confirmed to predict survival, treatment response, and diagnosis in patients with different OC, including epithelial ovarian cancer (EOC), high-grade serous ovarian carcinoma (HGSOC), and ovarian clear cell carcinoma (OCCC). Cramer et al. reported that NLR, PLR, and MLR could help us in the better identification of benign and malignant tumors [24] and in guiding changes in the chemotherapy regimen [25]. NLR is significantly increased in malignant ovarian cases and was the second most sensitive marker for predicting malignant tumors after cancer antigen 19–9 [26]. PLR values of ≥ 205.4 predicted incomplete remission in patients with OC (CR, accuracy is 71.6%) [27]. NLR and PLR can also be used to predict the distant metastasis of gynecological tumors [28]. Kokcu et al. reported that NLR and PLR showed an upward trend with the increase of OC stages [29]. Polat et al. confirmed through clinical studies that it was reasonable to observe the relationship between these hematological indexes and the prognosis of the disease. A high ratio may be an early sign of micrometastasis or dominant metastasis as well as a valuable prognostic index [30]. Meerwaldt et al. found that CRP and ALB could predict the prognosis of OC [31]. However, the study has suggested that PLR has no value in monitoring the postoperative status of patients with OC [32]; it was not significant in predicting the survival of patients with OC [33–36]. Topcu et al. showed that NLR was not an effective marker for predicting the malignant characteristics of pelvic masses [37]. Therefore, although these composite markers have been studied for many years, they are still not widely used in clinical practice compared with biomarkers such as serum CA125 and human epididymal protein 4. Currently, few guidelines or consensus statements emphasize the predictive value of these markers, and studies on these markers have not been well-summarized.

The inflammatory microenvironment can promote the occurrence and development of OC; however, the clinical studies on the predictive value of these blood inflammatory composite markers for OC have not been well-reviewed. The accuracy of prediction varies with the conditions of patients, time nodes, and other factors. The biomarkers for different subtypes of OC and the timelines for recording them have not been conclusively defined. This review critically summarizes the importance of blood inflammatory composite markers in predicting the occurrence and development of OC, elaborates on the advantages of using such biomarkers, and highlights future research directions to enhance their clinical applications.

We screened PubMed, EMBASE, Web of Science, and Cochrane Library for studies that may meet our criteria until June 2022. The search terms were set to “ovarian neoplasms” OR “ovarian cancer” OR “cancer of ovary” AND “neutrophil-to-lymphocyte ratio,” “platelet-to-lymphocyte ratio,” “monocyte-to-lymphocyte ratio,” “systemic inflammation Index,” “C-reactive protein albumin ratio,” “prognostic nutritional index” (Table S1). We eliminated the repeated studies by verifying the author’s name, institution, number of participants, and baseline data. We ensured that all the studies were included in Science Citation Index for high research quality. The results were limited to humans with ovarian cancer and the language was limited to English. All results were imported into EndNote (Vision X9.2).

The data were extracted independently by Chuan-long Zhang, Xiao-chen Jiang, and Yi Li. The main extracted information included the biomarker studied, author, year, conditions of study participants, cut-off values, low/ high (number), marker significance, and limitations of the study. We also extracted the area under receiver operating characteristics curve (AUC) values from the studies that used AUC analyses to determine the biomarker cut-off values. We only extracted independent prediction outcomes from the studies that reported survival prediction. In addition, we extracted the “limitation” of the study without any subjective factors. We focused on NLR and PLR, visually analyzed the forest map of the hazard ratios (HR) of these two markers with survival data in the included studies, and calculated the pooled HRs. The quality of data extraction was ensured by Yan Chen and Bo Pang (Table S2).

### Blood inflammatory composite markers based on complete blood count

#### NLR

**Fig.1:**
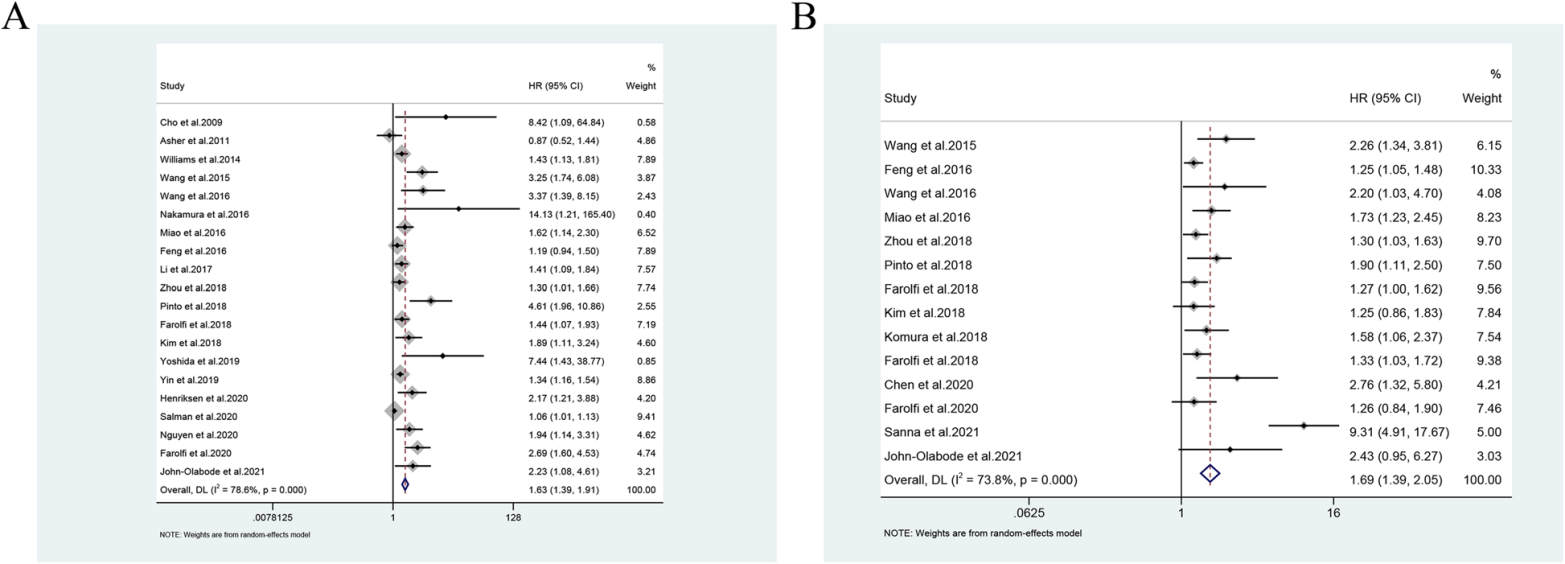
Forest plots for survival analysis in patients with ovarian cancer. **A** NLR for overall survival analysis. **B** NLR for progression-free survival. NLR, neutrophil-to-lymphocyte ratio; HR, hazard ratio; 95% CI, 95% confidence interval

We included 36 studies on NLR in our analysis, and 21 of them reported that NLR had an independent predictive value in predicting the survival of patients with OC. These studies included patients with EOC, HGSOC, and OCCC who had undergone surgery or chemotherapy. Cho et al. reported that preoperative high NLR predicted poor overall survival (OS) in patients with OC who underwent surgery [38], which was further confirmed by Li et al.[34, 36, 39–46]. Badora-Rybicka et al. observed NLR in 315 patients with EOC who received platinum-taxane chemotherapy after surgery, and found that high NLR could predict poor progression-free survival (PFS) but not OS [33]. Similar observations were reported by Feng et al. [47, 48]. Wang et al. found that the preoperative high NLR had predictive value for both poor OS and PFS in patients with OC [49–52]. Nakamura et al. suggested that higher NLR indicated higher mortality within 100 days of the failure of final-line chemotherapy [53]. Pinto et al. found that the increased NLR after surgery also predicted the poor OS of the patients [54]. Notably, similar observations were reported at different time nodes in these studies. Kim et al. noted the predictive value of NLR before treatment. Further, they also measured the dynamic changes in NLR during chemotherapy and confirmed that the increased NLR during chemotherapy was an independent predictor of PFS in patients with OC [55]. The pooled HR was 1.63 (95% CI: 1.39, 1.91; Fig. 1A) for the prediction of OS by NLR, and, the pooled HR was 1.69 (95% CI: 1.39, 2.05, Fig. 1B) for the prediction of PFS. The prediction value of NLR indicated its potential in predicting survival in patients with OC who underwent surgery. Overall, the pooled HRs showed that NLR could predict OS and PFS in patients with OC.

Several studies have focused on the importance of NLR in early diagnosis and in predicting treatment response and distant metastases of malignant tumors. Medina et al. showed that NLR was used to monitor the postoperative evolution of surgical patients, but could not be used to detect infectious complications [32]. Wang et al. stratified the value of NLR, which confirmed that NLR had a predictive value for chemotherapy response [49]. Several subsequent studies confirmed that high NLR before treatment or before surgery usually indicated poor surgical outcomes [42, 56, 57], poor efficacies of chemotherapy and immunotherapy [39, 43, 58, 59], or even drug resistance [60]. Inflammation could mediate drug resistance in cancer treatment [61]. In 2014 and 2015, Yildirim et al. demonstrated that preoperative NLR could be used for the early detection of ovarian malignant tumors, and preoperative NLR could predict the pathological diagnosis of adnexal masses [62, 63]. They concluded that the difference in preoperative NLR level could assist in the differential diagnosis of malignant and benign ovarian tumors; similar conclusions were reported in many other studies [26, 30, 64–67]. Chen et al. also confirmed the use of NLR in the differential diagnosis of OC and endometriosis [68]. Abu-Shawer et al. evaluated 264 patients with FIGO stage III and IV gynecological (endometrial, ovarian, and cervical) cancers and found that baseline NLR was a marker for predicting the presence of distant metastases [28] (Table 1). Currently, the cut-off values of NLR range from 0.89 to 6 with the most frequent values concentrated in the range from 2 to 4 (Table S3). However, Forget et al. reported similar values in healthy people (0.78 to 3.53) [69]. Therefore, the cut-value of NLR in predicting OC needs to be determined for clinical applications.

**Table 1:** Summary of the eligible studies on NLR in predicting survival treatment response, and diagnosis, in patients with ovarian cancer

Author, Year	Conditions of participants	Significance of marker
Cho et al. [38], 2009; Badora-Rybicka et al.[70], 2016; Wang et al. [49], 2016; Nakamura et al. [53], 2016; Miao et al. [50], 2016; Feng et al. [71], 2016; Li et al. [34], 2017; Farolfi et al. [39], 2018; Zhou et al. [51], 2018; Pinto et al. [54], 2018; Pinto et al. [54], 2018; Komura et al. [48], 2018; Kim et al. [55], 2018; Baert et al. [52], 2018; Ceran et al. [40], 2019; Yoshida et al. [36], 2019; Salman et al. [41], 2020; Henriksen et al. [43], 2020; Farolfi et al. [44], 2020; Nguyen et al. [42], 2020; John-Olabode et al. [45], 2021; Liontos et al. [46], 2021	**OCP:** received operations, with stage IIIC and IV received NACT, with recurrence treated with chemotherapy; **EOCP:** underwent elective surgery, receiving platinum-taxane CT after surgery, in FIGO stage III-IV treated with first-line CT or in CTB, with FIGO stage IIIC underwent PDS, received NACT, received their complete primary treatment; **HGSOCP:** underwent surgery (PDS); **OCCCP:** underwent surgery with stage I–II	Predicting survival: OS, PFS, mortality within 100 days of the failure of final‑line CT
Ashrafganjoe et al. [56], 2016; Farolfi et al. [39], 2018; Boland et al. [58], 2019; Henriksen et al. [43], 2020; Chen et al. [60], 2020; Nguyen et al. [42], 2020; Winarno et al. [59], 2021; Sastra et al. [57], 2022	**OCP:** recurrent and received CT; **EOCP:** underwent primary staging exploratory laparotomy, in FIGO stage III-IV treated with first-line CT or CTB; received ICB, underwent platinum CT after cytoreduction surgery; **HGSOCP:** underwent surgery (PDS); **OCCCP:** with IC-IV stage	Predicting treatment response: surgical outcome, efficacy of CT, early discontinuation of ICB; platinum resistance, 30-day postoperative morbidity
Yildirim et al. [62], 2014; Yildirim et al. [63], 2015; Seckin et al. [26], 2015; Polat et al. [30], 2016; Wu et al. [64], 2019; Eo et al. [65], 2018; Li et al. [66], 2021; Yun et al. [67], 2022	**OCP:** with documented benign and with malignant adnexal masses underwent primary surgery; patients with adnexal masses underwent surgical resection; patients had borderline, patients had benign, and patients had malignant mucinous ovarian tumors; women with sonographically detected ovarian tumor; OCP, patients with benign ovarian disease, and healthy controls; **EOCP:** underwent surgery; patients with benign ovarian masses and EOCP underwent surgery	Predicting diagnosis: malignant or not, malignant degree, histotypes, benign or borderline tumors

*NLR* Neutrophil-to-lymphocyte ratio, *OCP* Ovarian cancer patients, *EOCP*, Epithelial ovarian cancer, Patients, *HGSOCP*, High-grade serous ovarian carcinoma patients, *OCCCP*, Ovarian clear cell carcinoma patients, *PDS* Primary debulking surgery, *CT* Chemotherapy, *CTB* Chemotherapy with bevacizumab, *NACT* Neoadjuvant chemotherapy, *ICB* Immune checkpoint blockade, *CRS* Cytoreductive surgery, *OS* Overall survival, *PFS* Progression-free survival

### PLR

**Fig.2:**
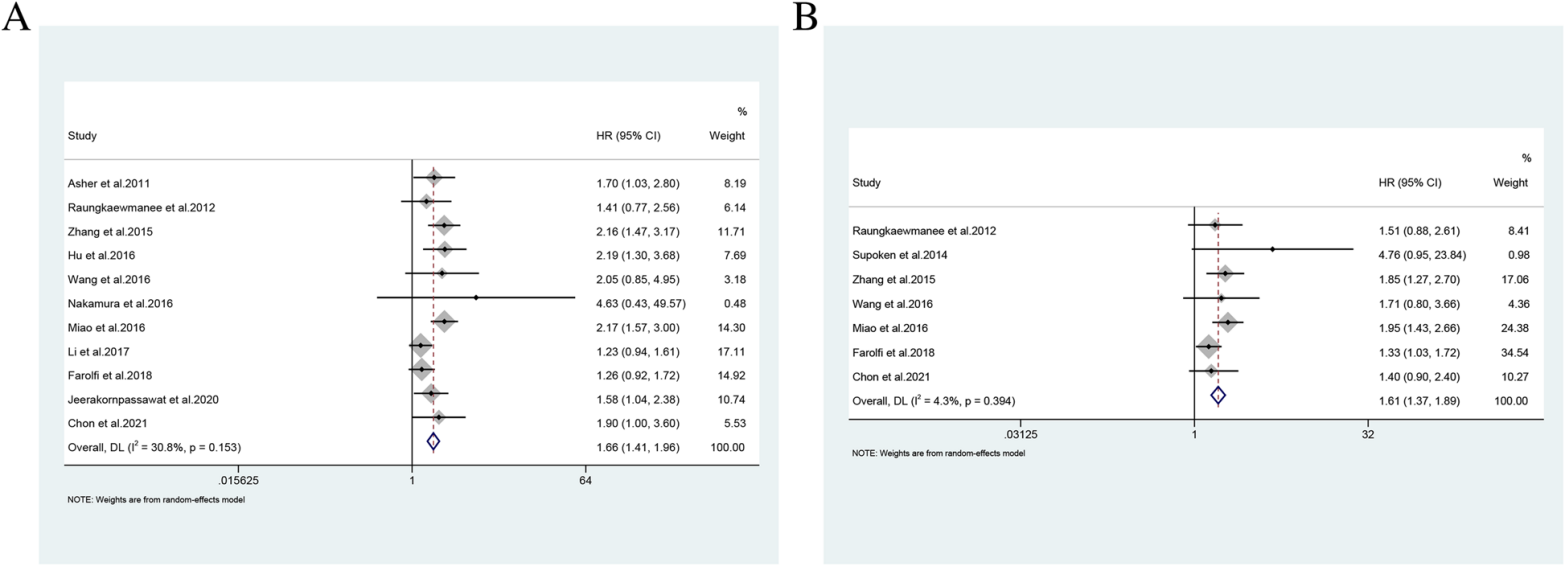
Forest plots for survival analysis in patients with ovarian cancer. **A** PLR for overall survival analysis. **B** PLR for progression-free survival. PLR, platelet-to-lymphocyte ratio; HR, hazard ratio; 95% CI, 95% confidence interval

We analyzed 22 studies on PLR and found that 10 of them focused on the predictive value of PLR for predicting survival in patients with OC. Viren Asher et al. suggested, for the first time, that PLR may a new independent prognostic marker for patients with OC. Preoperative high PLR predicted poor OS [72]. Several authors [40, 49, 73, 74] had come to this conclusion. Zhang et al. [75] and Miao et al. [50] showed that PLR was also valuable in predicting PFS in addition to OS in OC. Farolfi et al. [39] observed the predictive value of PLR for PFS, whereas Jammal et al. [62] and Ramón-Rodríguez et al. [76] determined its predictive value in predicting disease-free survival (DFS). Yoshida et al. reported that elevated PLR was not useful in predicting adverse OS and PFS in patients with early-stage OCCC [36]. Jeerakornpassawat et al. confirmed high PLR is a potential independent predictive factor for poor survival outcomes of patients with EOC, fallopian tube, and primary peritoneal cancer treated with platinum-based chemotherapy [77]. In these studies, PLR was measured before surgery or before chemotherapy. For the prediction of OS by PLR, the pooled HR was 1.66 (95% CI: 1.41–1.96) (Fig. 2A), and for the prediction of PFS, the pooled HR was 1.61 (95%CI: 1.37–1.89) (Fig. 2B). This suggested that high PLR predicted poor OS and PFS in patients with OC. PLR may be less affected by infection and autoimmune diseases than NLR because platelets are used to calculate this ratio.

PLR could well predict the staging and surgical outcomes of patients with OC after surgery [78]. Several researchers [56, 57] also showed that PLR could predict surgical outcome in patients with OC. Winarno et al. determined that post-operative PLR could predict the response of patients to chemotherapy [59]. Abu-Shawer et al. showed that baseline PLR was a marker for predicting the presence of distant metastases in patients with FIGO stage III and IV gynecological (endometrial, ovarian, and cervical) cancers [28]. Yildirim et al. reported that PLR could play a predictive role in the early diagnosis of OC and could effectively predict the pathological diagnosis of adnexal masses in OC [62, 63]. Several researchers [30, 65, 79] showed that preoperative PLR was a predictive marker for ovarian malignant tumors. Wang et al. [80], and Li et al. [66] found that preoperative PLR was a predictor of the recurrence of OC. Yun et al. showed that preoperative high PLR suggested that ovarian cancer was more likely to occur than benign or borderline tumors [67] (Table 2). However, the cut-off values of PLR were different in all the studies because of the differences in methods used to obtain these values (Table S2). The cut-off values were obtained through the receiver operating characteristic (ROC) curve, log-rank test, or referring to previous studies. Therefore, standardized universal cut-off values for the blood inflammatory composite markers, including PLR, are not defined. And we found that the results of some studies did not fully support the conclusions of his study [78].

**Table 2:** Summary of the eligible studies on PLR in predicting survival, treatment response, and diagnosis in patients with ovarian cancer

Author, Year	Conditions of participants	Significance of marker
Asher et al. [72], 2011; Zhang et al. [75], 2015; Wang et al. [49], 2016; Miao et al. [50], 2016; Hu et al. [73], 2016; Farolfi et al. [39], 2018; Ceran et al. [40], 2019; Jammal et al. 2020[81]; Chon et al. [74], 2021; Ramón-Rodríguez et al. [82], 2022	**OCP:** underwent surgery, with CRS followed by platinum-based CT **EOCP:** receiving platinum-based CT, in FIGO stage III-IV EOCP treated with first-line CT or in CTB, underwent surgery, with advanced stage; **HGSOCP:** underwent surgical resection of primary cancers; **Ovarian peritoneal carcinomatosis:** underwent CRS with HIPEC	Predicting survival: OS, PFS, and DFS
Ashrafganjoe et al. [56], 2016; Sastra et al. [57], 2022; Raungkaewmanee et al. [78], 2012; Winarno et al. [59], 2021	168 EOCP underwent primary staging exploratory laparotomy; 54 Indonesian EOCP underwent primary exploratory laparotomy; 166 EOCP underwent surgery; 95 EOCP underwent platinum CT after cytoreduction surgery	Predicting treatment response: surgical outcome, efficacy of CT
Yildirim et al. [62], 2014; Yildirim et al. [63], 2015; Ozaksit et al. [79], 2015; Polat et al. [30], 2016; Eo et al. [65], 2018; Wang et al. [80], 2021;Li et al. [66], 2021; Yun et al. [67], 2022	316 OCP with documented benign and 253 OCP with malignant adnexal masses underwent primary surgery; 306 patients with adnexal masses underwent surgical resection; 196 adolescent females with adnexal masses; 275 women with sonographically detected ovarian tumor; 229 EOCP; 43 OCP with recurrence; 207 patients with benign ovarian masses and 887 EOCP who underwent surgical resection; 630 patients with ovarian tumors	Predicting diagnosis: malignant or not, malignant degree, advanced-stage, recurrence, histotypes, benign or borderline tumors

*PLR* Platelet-to-lymphocyte ratio, *OCP* Ovarian cancer patients, *EOCP* Epithelial ovarian cancer patients, *HGSOCP* High-grade serous ovarian carcinoma patients, *CT* Chemotherapy, *CTB* Chemotherapy with bevacizumab, *CRS* Cytoreductive surgery, *OS* Overall survival, *PFS* Progression-free survival, *DFS* Disease-free survival

MLR and SII.

Few studies have reported the predictive value of MLR in patients with OC. Xiang et al. confirmed that preoperative MLR is a predictor of advanced stages, advanced pathologic grades, positive lymphatic metastasis, and OS in patients with OC [83]. Sastra et al. determined that higher preoperative MLR predicted worse surgical outcomes [57]. Guo et al. proposed a new diagnostic normal map model (including preoperative MLR) which predicted the degree of malignancy in patients with ovarian masses [84] (Table 3). MLR cut-off values ranged from 0.23 to 0.249 (Table S5).

**Table 3:** Summary of the eligible studies on MLR in predicting survival, treatment response, and diagnosis in patients with ovarian cancer

Author, Year	Conditions of participants	Significance of marker
Xiang et al. [83], 2017	**OCP**	Predicting survival: OS
Sastra et al. [57], 2022	**EOCP:** underwent primary exploratory laparotomy	Predicting treatment response: surgical outcome
Guo et al. [84], 2021	**OCP:** underwent surgery	Predicting diagnosis: malignant or not, malignant degree

*MLR* Monocyte-to-lymphocyte ratio, *OCP* Ovarian cancer patients, *EOCP* Epithelial ovarian cancer patients, *OS* Overall survival

Nie et al. found that preoperative high SII was an independent prognostic factor for poor OS and PFS in patients with OC [85]. Farolfi et al. [44] and Ramón-Rodríguez et al. [82] suggested that the value of high SII predicted OS in patients with OC. Farolfi et al. also showed that the baseline high SII independently indicated the poor efficacy of chemotherapy in patients with OC (Table 4). SII cut-off values ranged from 564.8 to 730 (Table S6).

**Table 4:** Summary of the eligible studies on SII in predicting survival, treatment response, and diagnosis in patients with ovarian cancer

Author, Year	Conditions of participants	Significance of marker
Nie et al. [85], 2019; Farolfi et al. [44], 2020; Ramón-Rodríguez et al. [82], 2022;	**OCP:** platinum-sensitive recurrent treated with a second-line therapy; **EOCP:** underwent primary surgery; **Ovarian peritoneal carcinomatosis:** underwent CRS with HIPEC	Predicting survival: OS, PFS
Farolfi et al. [39], 2018	**EOCP:** in FIGO stage III-IV treated with first-line CT or CTB	Predicting treatment response: efficacy of CT

*SII* Systemic inflammation index, *OCP* Ovarian cancer patients, *EOCP* Epithelial ovarian cancer patients, *HIPEC* Hyperthermic intraperitoneal chemotherapy, *CRS* Cytoreductive surgery, *OS*, Overall survival, *PFS* Progression-free survival, *CT* Chemotherapy, *CTB* Chemotherapy with bevacizumab

### Blood inflammatory composite markers based on blood protein

#### CAR and PNI

Liu et al. [35] and Komura et al. [86] conducted clinical studies on the CAR-based prediction of survival in patients with OC and found that high CAR before treatment was an independent marker for poor OS and disease-specific survival (DSS) (Table 5).

**Table 5:** Summary of the eligible studies on CAR in predicting survival, treatment response, and diagnosis in patients with ovarian cancer

Author, Year	Conditions of participants	Significance of marker
Liu et al. [35], 2017; Komura et al. [86], 2021	**OCP:** underwent surgery; **EOCP:** accepted treatment	Predicting survival: OS, DSS

*CAR* C-reactive protein-to-albumin ratio, *OCP* Ovarian cancer patients, *EOCP* Epithelial ovarian cancer patients, *OS* Overall survival, *DSS* Disease-specific survival

Miao et al. [87] suggested that PNI was an independent prognostic indicator for OS and PFS in OC patients. Some authors [88–90] pointed out that the decrease in PNI was an independent predictor of poor OS, but not PFS. PNI was an independent predictor of OS in patients in OC as a continuous variable [47]. Komura et al. showed that PNI was an independent prognostic factor for poor PFS and DSS [91]. Xing et al. found that postoperative PNI was an independent predictor of 1-year recurrence [90]. In patients with advanced OC, the increased PNI was not correlated with poor OS, but it helped predict the early stage of OC, and the predictive AUC for the efficacy of chemotherapy was 69%, which was an acceptable predictive value [92]. Miao et al. [73] also supported the observation that high PNI was an independent predictor of chemotherapy efficacy [87] (Table 6).

**Table 6:** Summary of the eligible studies on PNI in predicting survival, treatment response, and diagnosis in patients with ovarian cancer

Author, Year	Conditions of participants	Significance of marker
Miao et al. [87], 2016; Zhang et al. [88], 2017; Feng et al. [47], 2018; Komura et al. [91], 2019; Yoshikawa et al. [89], 2020; Xing et al. [90], 2022	**OCP:** in FIGO III stage underwent CRS followed by platinum-based CT; underwent surgery **EOCP:** receiving platinum-based CT, with advanced stage; **HGSOCP:** with primary staging underwent DS; **OCCCP:** with stage I–II (FIGO 2014) undergoing primary surgery	Predicting survival: OS, PFS, DSS
Karakaş et al. [92], 2022; Miao et al. [87], 2016	**OCP:** with early-stage underwent surgery; **EOCP:** receiving platinum-based CT	Predicting treatment response: efficacy of CT
Xing et al. [90], 2022	**OCP:** underwent surgery	Predicting diagnosis: 1-year recurrence

*PNI* Prognostic nutritional index, *OCP* Ovarian cancer patients, *EOCP* Epithelial ovarian cancer patients, *OCCCP* Ovarian clear cell carcinoma patients, *CT* Chemotherapy, *DS* Debulking surgery, *OS*, overall survival, *PFS*, Progression-free survival, *DSS* Disease-specific survival

The two studies on CAR reported variable cut-off values, which may not be informative (Table S7). The cut-off values of PNI were between 42.9 and 50.4 (Table S8). Presently, there is no gold standard nutritional assessment method to predict the prognosis of patients with OC. It was difficult to compare the advantages and disadvantages of these two markers. We believed that the dynamic changes in these markers were related to the level of systemic inflammation and nutritional status, and the values changed more often because of the response to treatment. Therefore, we recommend that further research should focus on predicting the treatment response of patients with OC to provide guidance for patients and doctors on treatment choices. The dynamic values of these markers may be more significant than the baseline values.

## Conclusion and Perspective

**Fig.3:**
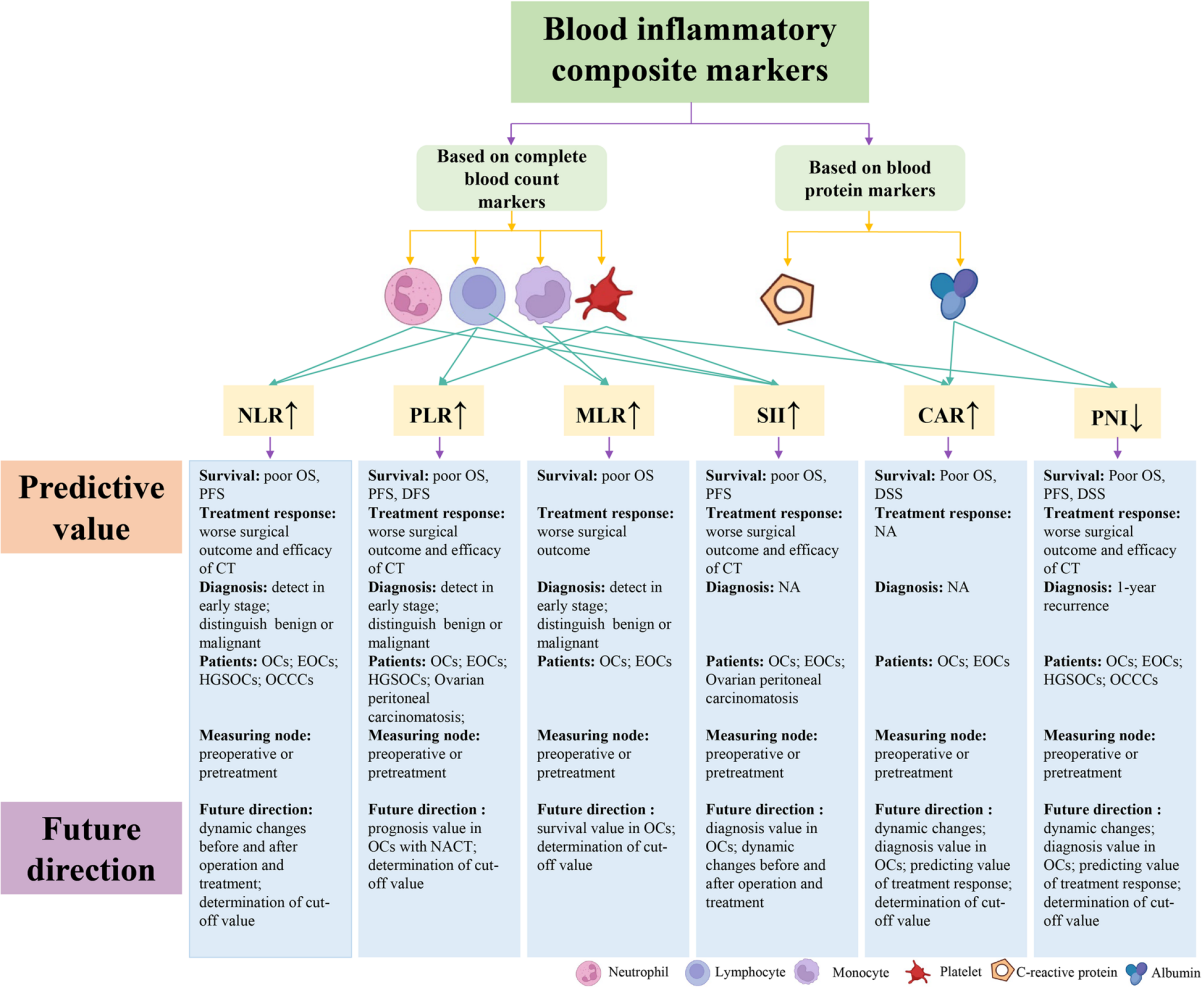
Overview of predictive values of blood inflammatory composite markers in ovarian cancer and future research directions. NLR, neutrophil-to-lymphocyte ratio; PLR, platelet-to-lymphocyte ratio; MLR, monocyte-to-lymphocyte ratio; SII, systemic inflammation index; CAR, C-reactive protein-to-albumin ratio; PNI, prognostic nutritional index; OCP: ovarian cancer patients; EOCP: epithelial ovarian cancer patients; HGSOCP: high-grade serous ovarian carcinoma patients; OCCCP: ovarian clear cell carcinoma patients; CT, chemotherapy; NACT, neoadjuvant chemotherapy; OS, overall survival; PFS, progression-free survival; DFS, disease-free survival; DSS, disease-specific survival

NLR, PLR, MLR, SII, CAR, and PNI have notable predictive value in predicting survival, treatment response, and diagnosis in patients with OC. Our review further emphasizes their predictive values. The analysis of these biomarkers is advantageous because of low cost, easy access, and less trauma, which can be beneficial for patients. Further, these blood inflammatory composite markers may have the potential to identify patients with high-risk OC as candidates for more intensive treatment in addition to standard treatment. The current predicted values and future research directions for these composite markers are illustrated in Fig. 3. We reviewed AUC in the existing clinical studies and found that these biomarkers had an acceptable predictive performance. However, the gap between clinical research and clinical applications of composite markers still exists because of two major reasons. First, their cut-off values have not been determined. Second, these values are easily affected by several confounding factors. Therefore, a prediction model is required to establish and verify the cut-off values of these composite biomarkers. A prospective multicenter study, including large sample size and assured follow-up, is required to generate data for building such a model.

Further, the dynamic changes in these markers may be of greater value and worthy of further studies. The differences in the timing of measuring these biomarkers, such as at diagnosis and before, during, and after treatment may have different implications for guiding clinical therapies. Even though neoadjuvant chemotherapy (NACT) for OC had always been controversial, its clinical efficacy is undeniable [93, 94]. However, none of the patients experienced NACT in the study population in which PLR was evaluated as a composite marker. Therefore, we have selected patients on NACT as our target group in our future study. The diagnostic values of the three markers SII, CAR, and PNI have not been determined; this is also a good direction for research. Presently, few researchers have worked on predicting the response to immunotherapy using such composite markers, and this aspect can be explored further. In the context of the study population, the number of studies focused on patients with EOC is higher than those focused on patients with OCCC and HGSOC. A comparison of the values of the same biomarker for different ovarian cancer subtypes would be immensely beneficial in guiding clinicians.

We extracted the limitations of the included studies without subjective factors and found that they had some common limitations: retrospective study design, small sample size, single-centric, and influence of confounding factors. Only one of the included studies had a prospective design [95]. These limitations biased their findings and decreased the reliability of their conclusions. In addition, we did not include studies on blood markers that can guide the prognosis of ovarian cancer, such as F-NLR [96], prognostic inflammation score [49], NLPN score (recurrent neutrophil–lymphocyte ratio × number of previous regimens) [89], and multiplication of neutrophil and monocyte counts [97], which may also be of great predictive value in OC. Clinicians urgently require better and cheaper biomarkers to predict the diagnosis and prognosis of patients with OC. However, the repetition of these studies is not advisable unless they have clinical applications. We recommend personalized medical research, higher-quality multicenter clinical studies, and more case observations for blood inflammatory composite markers to develop a comprehensive strategy for using these markers in predicting different parameters in patients with OC.

## Supplementary Information

**Additional file 1**.

**Additional file 2**.

**Additional file 3**.

**Additional file 4**.

**Additional file 5**.

**Additional file 6**.

**Additional file 7.**

**Additional file 8.**

## Data Availability

All data are included in the manuscript.

## Abbreviations

AUC: Area under the Curve
ROC: Receiver Operating Characteristic
N: Number of patients
NA: Not available
NLR: Neutrophil-to-lymphocyte ratio
PLR: Platelet-to-lymphocyte ratio
MLR: Monocyte-to-lymphocyte ratio
SII: Systemic inflammation index
CRP: C-reactive protein
ALB: Albumin
CAR: C-reactive protein-to-albumin ratio
PNI: Prognostic nutritional index
HR: Hazard ratio
95% CI: 95% Confidence interval
OC: Ovarian cancer
EOC: Epithelial ovarian cancer
HGSOC: High-grade serous ovarian carcinoma
OCCC: Ovarian clear cell carcinoma
OCP: Ovarian cancer patients
EOCP: Epithelial ovarian cancer patients
HGSOCP: High-grade serous ovarian carcinoma patients
OCCCP: Ovarian clear cell carcinoma patients
PDS: Primary debulking surgery
DS: Debulking surgery
CT: Chemotherapy
CTB: Chemotherapy with bevacizumab
NACT: Neoadjuvant chemotherapy
ICB: Immune checkpoint blockade
HIPEC: Hyperthermic intraperitoneal chemotherapy
CRS: Cytoreductive surgery
OS: Overall survival
PFS: Progression-free survival
DFS: Disease-free survival
DSS: Disease-specific survival
