# Supplementary material for: Independent predictive value of blood inflammatory composite markers in ovarian cancer: recent clinical evidence and perspective focusing on NLR and PLR
Source: J Ovarian Res. 2023 Feb 9;16:36. doi: 10.1186/s13048-023-01116-2 (PMC9912515; doi:10.1186/s13048-023-01116-2)
Supplement: Supplementary file 2 — Additional file 2. [file 13048_2023_1116_MOESM2_ESM.docx]

**Fig.1** Forest plots for survival analysis in patients with ovarian cancer. **A** NLR for overall survival analysis. **B** NLR for progression-free survival. NLR, neutrophil-to-lymphocyte ratio; HR, hazard ratio; 95% CI, 95% confidence interval.

**Fig.2** Forest plots for survival analysis in patients with ovarian cancer. **A** PLR for overall survival analysis. **B** PLR for progression-free survival. PLR, platelet-to-lymphocyte ratio; HR, hazard ratio; 95% CI, 95% confidence interval.

**Fig.3** Overview of predictive values of blood inflammatory composite markers in ovarian cancer and future research directions. NLR, neutrophil-to-lymphocyte ratio; PLR, platelet-to-lymphocyte ratio; MLR, monocyte-to-lymphocyte ratio; SII, systemic inflammation index; CAR, C-reactive protein-to-albumin ratio; PNI, prognostic nutritional index; OCP: ovarian cancer patients; EOCP: epithelial ovarian cancer patients; HGSOCP: high-grade serous ovarian carcinoma patients; OCCCP: ovarian clear cell carcinoma patients; CT, chemotherapy; NACT, neoadjuvant chemotherapy; OS, overall survival; PFS, progression-free survival; DFS, disease-free survival; DSS, disease-specific survival.
